# Old divergence and restricted gene flow between torrent duck (*Merganetta armata*) subspecies in the Central and Southern Andes

**DOI:** 10.1002/ece3.5538

**Published:** 2019-08-15

**Authors:** Luis Alza, Philip Lavretsky, Jeffrey L. Peters, Gerardo Cerón, Matthew Smith, Cecilia Kopuchian, Andrea Astie, Kevin G. McCracken

**Affiliations:** ^1^ Department of Biology University of Miami Coral Gables FL USA; ^2^ División de Ornitología CORBIDI Lima Peru; ^3^ Institute of Arctic Biology Department of Biology and Wildlife University of Alaska Fairbanks AK USA; ^4^ Department of Biological Sciences University of Texas at El Paso El Paso TX USA; ^5^ Department of Biological Sciences Wright State University Dayton OH USA; ^6^ Laboratorio de Zoología‐CRUB Universidad Nacional del Comahue Bariloche Argentina; ^7^ Centro de Ecología Aplicada del Litoral (CECOAL‐CONICET) Corrientes Argentina; ^8^ División Ornitología Museo Argentino de Ciencias Naturales (MACN‐CONICET) Buenos Aires Argentina; ^9^ Instituto Argentino de Investigaciones de las Zonas Áridas (CCT Mendoza‐CONICET) Mendoza Argentina; ^10^ Rosenstiel School of Marine and Atmospheric Sciences University of Miami Coral Gables FL USA; ^11^ University of Alaska Museum University of Alaska Fairbanks Fairbanks AK USA

**Keywords:** Andes, gene flow, genetic diversity, Merganetta armata, population structure, time since divergence

## Abstract

**Aim:**

To investigate the structure and rate of gene flow among populations of habitat‐specialized species to understand the ecological and evolutionary processes underpinning their population dynamics and historical demography, including speciation and extinction.

**Location:**

Peruvian and Argentine Andes.

**Taxon:**

Two subspecies of torrent duck (*Merganetta armata*).

**Methods:**

We sampled 156 individuals in Peru (*M. a. leucogenis;* Chillón River, *n* = 57 and Pachachaca River, *n* = 49) and Argentina (*M. a. armata;* Arroyo Grande River, *n* = 33 and Malargüe River, *n* = 17), and sequenced the mitochondrial DNA (mtDNA) control region to conduct coarse and fine‐scale demographic analyses of population structure. Additionally, to test for differences between subspecies, and across genetic markers with distinct inheritance patterns, a subset of individuals (Peru, *n* = 10 and Argentina, *n* = 9) was subjected to partial genome resequencing, obtaining 4,027 autosomal and 189 Z‐linked double‐digest restriction‐associated DNA sequences.

**Results:**

Haplotype and nucleotide diversities were higher in Peru than Argentina across all markers. Peruvian and Argentine subspecies showed concordant species‐level differences (Φ_ST_ mtDNA = 0.82; Φ_ST_ autosomal = 0.30; Φ_ST_ Z chromosome = 0.45), including no shared mtDNA haplotypes. Demographic parameters estimated for mtDNA using IM and IMa2 analyses, and for autosomal markers using *∂a∂i* (isolation‐with‐migration model), supported an old divergence (mtDNA = 600,000 years before present (ybp), 95% HPD range = 1.2 Mya to 200,000 ybp; and autosomal *∂a∂i* = 782,490 ybp), between the two subspecies, characteristic of deeply diverged lineages. The populations were well‐differentiated in Argentina but moderately differentiated in Peru, with low unidirectional gene flow in each country.

**Main conclusions:**

We suggest that the South American Arid Diagonal was preexisting and remains a current phylogeographic barrier between the ranges of the two torrent duck subspecies, and the adult territoriality and breeding site fidelity to the rivers define their population structure.

## 1 INTRODUCTION

Establishing how populations are structured is fundamental for understanding evolutionary processes (Hartl & Clark, 2007; Hey & Machaco, 2003; Ma, Ji, & Zhang, 2015; Wright, 1969). As populations subdivide through time, variation in the rate of genetic drift and gene flow, as well as selective pressures, will define the genetic diversity, divergence, or extinction of each species (Bowler & Benton, 2005; Frankham, 2005; Hey, 2010; Lenormand, 2002; Ma et al., 2015; Shaffer, 1981; Slatkin, 1987). Among types of species, habitat specialists are particularly prone to increased levels of population structure as a result of isolation on patchy habitats (Kawecki & Ebert, 2004; Kawecki et al., 2012; Nei, 2013; Orr, 2005). In addition, patchy habitats often harbor small populations with low genetic diversity, which may reduce the effectiveness of selection and contribute to high rates of local extinction (Charlesworth, 2010; Hartl & Clark, 2007). The vulnerability to extinction by isolated populations also occurs because of lack of suitable habitat, environmental pressures (e.g., development, predation, climate change), and/or stochastic perturbations (i.e., survival and reproductive success, habitat variation, genetic drift, catastrophes) (Callaghan, 1997; Fahrig, 2013; Naranjo & Ávila, 2003; Shaffer, 1981; Soulé & Mills, 1998). Therefore, characterizing population structure and the rate of gene flow among populations of habitat specialists is crucial for understanding the ecological and evolutionary processes underpinning population dynamics and historical demography, including speciation and extinction (Hartl & Clark, 2007; Lenormand, 2002; McCauley, 1991; Neigel, 1997; Slatkin, 1993).

The torrent duck (*Merganetta armata* Gould, 1842) is a specialized waterfowl of fast‐flowing whitewater mountain rivers that occupies 12% of the approximately 1,142 rivers (between 500 and 4,100 m a.s.l.) on both slopes of the world's longest mountain range, the Andes (eBird, 2016; Fjeldså & Krabbe, 1990). Across their range, torrent ducks display plumage coloration and body size differences (Fjeldså & Krabbe, 1990; Gutiérrez‐Pinto et al., 2014; Johnsgard, 2010; Naranjo & Ávila, 2003), resulting in at least three described subspecies: *M. a. colombiana* (Venezuela to Ecuador), *M. a. leucogenis* (Peru, Bolivia, Argentina and Chile), and *M. a. armata* (Argentina and Chile). Here, we focus on the two subspecies distributed in the highest regions of the Andes (Brack, 2000; Burkart, Bárbaro, Sánchez, & Gómez, 1999; Capitonio, Faccenna, Zlotnik, & Stegman, 2011; Fjeldså & Krabbe, 1990): specifically, the subspecies *M. a. leucogenis* (300–520 g) in the Central Andes of Peru, and the larger (360–580 g) and darker‐colored subspecies *M. a. armata* in the Southern Andes of Argentina (Johnsgard, 2010). Although the subspecies classification is supported by morphological differences, genetic information that supports this classification is lacking. Furthermore, it is unclear whether these subspecies were differentiated by ancient vicariant event (e.g., uplift of the Andes, desertification etc.) or a contemporary founder event (e.g., post glaciation). Additionally, the extent of gene flow among many patchily distributed riverine populations across the Andes remains unknown.

In the rivers occupied by torrent ducks, the adults show a high breeding site fidelity (Alza et al., 2017; Cardona & Kattan, 2010) to territories that reportedly range from 0.7 to 2 km during the breeding season (Cerón & Trejo, 2009; Colina, 2010; Eldridge, 1986; Johnsgard, 2010; Moffet, 1970; Naranjo & Ávila, 2003; Ubeda, Cerón, & Trejo, 2007). However, immature and adult individuals have been reported moving away from rivers likely searching for new habitats (i.e., four opportunistic records of male‐biased dispersal; Cerón & Capllonch, 2016). Thus given that torrent duck pairs are territorial with a high rate of river‐specific site fidelity, we expect torrent ducks to be more genetically similar among the nearest rivers but isolated by distance among rivers depending on the dispersal capacity of the ducks (Hanski & Gilpin, 1991; Harrison & Hastings, 1996; Nielsen & Slatkin, 2013; Wright, 1943). Alternatively, different riverine subpopulations may follow a metapopulation model (Hanski & Gaggiotti, 2004; Levins, 1968), in which populations or subpopulations isolated inside watersheds are still connected by limited gene flow, that would play a key role in recolonization (sporadic dispersal) after the extinction of a subpopulation.

Here, we reconstruct population structure for mitochondrial DNA (mtDNA) and thousands of nuclear markers isolated using partial genome resequencing (i.e., double‐digest restriction site‐associated DNA sequencing (ddRAD‐Seq)), attained from altitudinal sampling transects in Peru and Argentina. Specifically, we estimated the time since divergence and gene flow between the two subspecies, and also characterized population structure and gene flow between riverine populations within subspecies. Furthermore, we calculated genetic diversity across the different populations to infer whether specific riverine populations have experienced demographic processes such as population expansion or contraction. Because the spatial linear configuration of the rivers restricts suitable habitat and constrains the population, we also examined evidence for altitudinal clines in mtDNA haplotype frequency. Finally, this genetic characterization across altitudinal gradients permits us to explore whether genetic variation is associated with recently described morphological and physiological differences of torrent ducks at high elevations, including changes in body size (Gutiérrez‐Pinto et al., 2014), function of key enzymes (Dawson et al., 2016), changes in insulative properties of feathers (Cheek, Alza, & McCracken, 2018), and changes in the hypoxic ventilatory response (Ivy et al., 2019).

## 2 MATERIALS AND METHODS

### 2.1 Sampling and DNA extraction

**Figure 1:**
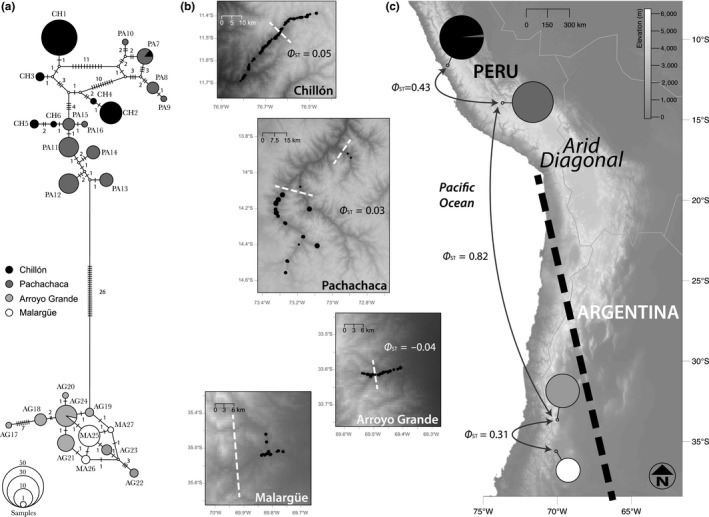
(a) Phylogenetic network of the 27 mtDNA haplotypes (634 base pairs of the CR mtDNA) found among four rivers for torrent ducks (*Merganetta armata*) in the Andes of Peru and Argentina, based on the statistical parsimony procedure implemented in TCS. Circle sizes are proportional to haplotype frequency (see inset, lower left); missing intermediate haplotypes are shown as small open dots. (b) River network morphology and sizes with the spatial distribution of the 156 individual of torrent ducks (*M. armata*), black dots, sampled along altitudinal gradients. Dot sizes represent different mtDNA haplotypes. These haplotypes do not present spatial segregation, Φ_ST_ values, along altitude. White dash lines mark the 2,500 m of elevation in each river system. Chillón River runs west, and Pachachaca, Arroyo Grande and Malargüe rivers run east. (c) Geographic distribution of the two rivers in Peru (*M. a. leucogenis*) and two rivers in Argentina (*M. a. armata*) along the Andes. Circle sizes represent the number of individuals sampled per watershed population, and color represents a haplogroup by origin associated to each river. Black dash line provides a reference of the Arid Diagonal spatial distribution

Blood samples were collected from 156 captured and released torrent ducks, during the 2010–2011 dry seasons, from two rivers in the western and eastern slopes of the Central Andes of Peru (Chillón River, *n* = 57 and Pachachaca River, *n* = 49; Table 1 and Figure 1b,c), and two rivers in the eastern slope of the southern Andes of Argentina (Arroyo Grande River, *n* = 32 and Malargüe River, *n* = 18; Table 1; Figure 1b,c). Each river was surveyed along an altitudinal transect (maximum range, 900–4,000 m) using an active mist net method by which torrent ducks were driven to nets (Alza et al., 2017). Blood samples (3 ml of whole blood per individual) were stored in liquid N_2_ in the field before being placed in long‐term storage at −80°C. Blood samples are archived at the University of Alaska Museum (Fairbanks, Alaska), Museo Argentino de Ciencias Naturales “Bernadino Rivadavia” (Buenos Aires, Argentina), and CORBIDI (Lima, Peru). DNA was extracted using a DNeasy Blood & Tissue kit and following the manufacturer's protocols (Qiagen). Extractions were quantified using a NanoDrop 2000 Spectrophotometer (Thermo Fisher Scientific Inc.) to ensure a minimum concentration of 20 ng/µl.

**Table 1:** The country, latitudinal distribution, physical characteristics, and elevation range of the four rivers where the two torrent duck (*Merganetta armata*) subspecies were sampled in the Andes of Peru and Argentina

Country	River	Latitude (°)	Watershed area (ha)	River + Tributary length (km)	Elevation range (m)
PERU	Chillón	11.5	161,280	87.08	1,000–4,000
	Pachachaca	13.5	671,844	326.80	2,700–3,100
ARGENTINA	Arroyo Grande	33.5	53,016	42.63	1,800–3,200
	Malargüe	35.5	69,652	37.59	1,600–1,900

### 2.2 Mitochondrial DNA

A 634‐base‐pair (bp) fragment (domains I and II) of the mtDNA control region (CR) was amplified and sequenced using specific primers for torrent ducks L100 (5′‐CATACATTTATGCGCCCCATAC‐3′) and H774 (5′‐CCATACACGCCAACCGTCTC‐3′) that prevented amplification of a known nuclear copy (K. G. McCracken, unpublished). PCR otherwise followed standard protocols (McCracken, Johnson, & Sheldon, 2001). Sequences were edited using sequencer v.4.7 (Gene Codes Corporation) and aligned by eye using se‐al v.2.0a11 (Rambaut, 2007). All sequences are deposited in GenBank (Accession Numbers MN196318:MN196473).

### 2.3 ddRAD‐Seq library preparation

While mtDNA was obtained across all samples, for more extensive genomic analysis of the two subspecies, a subset from Chillón River in Peru (*n* = 10) and Malargüe River in Argentina (*n* = 9) were subjected to partial genome resequencing via ddRAD‐Seq. Sample preparation for ddRAD‐Seq followed the double‐digest protocol outlined in DaCosta and Sorenson (2014). In short, ~1 μg of genomic DNA was digested using 10 U of each *Sbf*I and *Eco*RI restriction enzymes. Adapters containing sequences compatible for Illumina TruSeq reagents and barcodes for de‐multiplexing reads were ligated to the sticky ends generated by the restriction enzymes. The adapter‐ligated DNA fragments were then size‐selected for 300–450 bp using gel electrophoresis (2% low‐melt agarose) and purified using a MinElute Gel Extraction Kit (Qiagen). Size‐selected fragments were then PCR amplified with Phusion High‐Fidelity DNA Polymerase (Thermo Scientific), and the amplified products were cleaned using AMPure XP magnetic beads (Beckman Coulter, Inc.). Quantitative PCR using a Library Quantification Kit for Illumina (KAPA Biosystems) was used to measure the concentration of purified PCR products, and samples with compatible barcode combinations were pooled in equimolar concentrations. A multiplexed library was sequenced on an Illumina HiSeq 2,500 using single‐end 150 bp chemistry at the Tufts University Core Genomics Facility. Raw Illumina reads are deposited in NCBI's Sequence Read Archive (SRA data: SRP215991).

### 2.4 Bioinformatics of ddRAD‐Seq data

Raw Illumina reads were processed using a computational pipeline described by DaCosta and Sorenson (2014; http://github.com/BU-RAD-seq/ddRAD-seq-Pipeline). First, reads were assigned to individual samples based on barcode sequences using the *ddRADparser.py* script. Reads per sample were then collapsed into identical clusters using the *CondenseSequences.py* script with low‐quality reads (i.e., sequences that failed to cluster with any other reads (–id setting of 0.90) and with an average per‐base Phred score <20) filtered out with the *FilterSequences.py* script. Condensed and filtered reads from all samples were then concatenated and clustered with an –id setting of 0.85 in uclust. muscle v.3 (Edgar, 2004) was used to align and cluster reads, and samples within each aligned cluster were genotyped using the *RADGenotypes.py* script. Homozygotes and heterozygotes were identified based on thresholds outlined in DaCosta and Sorenson (2014; Lavretsky et al., 2015), with individual genotypes falling into three categories: “missing” (no data), “good” (unambiguously genotyped), and “flagged” (recovered heterozygous genotype, but with haplotype counts outside of acceptable thresholds or with >2 alleles detected). Loci with <20% missing genotypes and ≤6 flagged genotypes were retained for downstream analyses. Moreover, alignments with end gaps due to indels, ≥2 polymorphisms in the last five base pairs, and/or a polymorphism in the *Sbf*I restriction site were either automatically trimmed or flagged for manual inspection in geneious (Biomatters Inc.). Manual editing increased the total number of retained markers by ~7%, while reducing bias resulting from discarding loci with indels or high levels of polymorphisms. Finally, output files for downstream analyses were created with custom python scripts (Lavretsky et al., 2016) that incorporated sequencing depth. To limit any biases due to sequencing error and/or low depth, alleles were called “missing” unless they met our thresholds of a minimum of 5X coverage for homozygotes, and thus at least 10X coverage for heterozygotes to call both alleles with >99% predicted sequence accuracy.

Finally, autosomal and Z chromosome‐linked loci were identified as described in Lavretsky et al. (2015), with assignments based on differences in sequencing depth and homozygosity between males and females. Because females have only one Z chromosome, Z‐linked markers in females are expected to appear homozygous and be recovered at about half the sequencing depth of males.

### 2.5 Summary statistics

For mtDNA, we calculated summary statistics between rivers including haplotype diversity, nucleotide diversity (π/site), Tajima's *D*, and Φ_ST_ in arlequin v. 3.5.1.2 (Excoffier & Lischer, 2010). Tajima's *D* was calculated to test for the departure from neutrality in scenarios characterized by an excess of rare alleles. Negative values for this test statistic may indicate a population evolving under nonrandom processes such as directional selection or recent demographic expansion, whereas positive values may be indicative of population decline or balancing selection. For autosomal and Z chromosome‐linked markers, Tajima's *D* estimates were calculated in the R (http://cran.r-project.org/) program “pegas” v.0.10 (Paradis, 2010). Analysis of molecular variance (AMOVA) was run to examine genetic differentiation within and among the four rivers sampled from the two countries. Population pairwise Φ_ST_ was estimated under the Tamura and Nei (1993) model of nucleotide substitution. For ddRAD‐Seq autosomal and Z chromosome‐linked markers, pairwise Φ_ST_ (i.e., "nuc.F_ST") and nucleotide diversity (i.e., “nuc.div.within”) estimates were calculated in the R program “PopGenome” (Pfeifer, Wittelsbürger, Ramos‐Onsins, & Lercher, 2014); indel positions were excluded from analyses.

### 2.6 Population structure

For mtDNA, the population structure was visualized by reconstructing a haplotype network using tcs v.1.21 (Clement, Posada, & Crandall, 2000). tcs illustrates all connections that have a 95% probability of being the most parsimonious. Networks are more appropriate for intraspecific gene genealogies than rooted tree algorithms because population genealogies are often multifurcated, descendant genes coexist with persistent ancestral sequences and in the case of nuclear DNA recombination events produce reticulate relationships, where traditional phylogenetic trees treat all sequences as terminal taxa (Posada & Crandall, 2001).

Analysis of population subdivision for ddRAD‐Seq data was done using two methods. First, we used a principal component analysis (PCA) as implemented in the adegenet R program (i.e., “dudi.pca”; Dray & Dufour, 2007; also see Jombart, 2008). Next, maximum likelihood‐based individual assignment probabilities were calculated in admixture (Alexander & Lange, 2011; Alexander, Novembre, & Lange, 2009). To do so, biallelic single‐nucleotide polymorphisms (SNPs) for each autosomal and Z‐linked (males only) cluster were formatted for admixture analysis and then processed through PLINK (Purcell et al., 2007) following steps outlined in Alexander, Novembre, and Lange (2012). For each admixture analysis, a 10‐fold cross‐validation was performed, with a quasi‐Newton algorithm employed to accelerate convergence (Zhou, Alexander, & Lange, 2011). For each number of populations (*K* = 1–5) tested, we used a block‐relaxation algorithm for the point estimation, with analyses terminated once the change (i.e., delta) in the log‐likelihood of the point estimations increased by <0.0001. Final outputs were based on admixture proportions (*Q* estimates; the log‐likelihood of group assignment) per individual. All analyses were performed without a priori assignments.

### 2.7 Historical population demography of mtDNA

For mtDNA, we used the coalescent genealogy sampler softwares, im and ima2, which implement isolation‐with‐migration models (Hey, 2005, 2010; Hey & Nielsen, 2004; Nielsen & Wakeley, 2001). These models use a Bayesian Markov chain Monte Carlo (MCMC) method that simultaneously estimates demographic parameters associated with the genetic divergence between populations. Using IMa2, we assumed a 4‐population genealogy of two subspecies with two populations each and estimated: effective population sizes (Θ) of each of the extant populations, the ancestral population size (Θ) prior to population divergence, levels and direction of gene flow (immigration) into each population (*m*) scaled to the mutation rate, and time since divergence for the populations (*t*) (Figure 3a). The IMa2 analysis was run in triplicate, each with 20 Metropolis‐coupled chains of five million steps using a geometric heating scheme and a burn‐in period of 500,000 steps. Priors were defined as follows: Θ = 150, *m* = 10, and *t* = 50, based on available information of density, and previous runs of the isolation‐with‐migration model.

Meanwhile, we used the two‐population im model to estimate levels and direction of gene flow (*m*) scaled to the mutation rate, and time since population divergence (*t*) between the two subspecies inhabiting Peru and Argentina, respectively (Figure 4a). The divergence parameter, *t,* is scaled to the neutral mutation rate and can be converted to time in years with an accurate estimate of the mutation rate (Hey & Nielsen, 2004). For the mtDNA, we used the point estimate of the substitution rate and confidence intervals published by Peters, Gretes, and Omland (2005): 4.8 × 10^–8^ substitutions/site/year [95% confidence interval (CI) = 3.1 – 6.9 × 10^–8^ substitutions/site/year]. The im analysis was run in triplicate to check for consistency among runs, each with 20 Metropolis‐coupled chains of three million steps using a geometric heating scheme and a burn‐in period of 500,000 steps. Priors were defined as follows: Θ_PERU_ = 80, Θ_ARGENTINA_ = 60, Θ_ANCESTRAL_ = 150, *m*_PER.‐ARG._ = 10, *m*_ARG.‐PER._ = 10, *t* = 40, and *s* = 0–1.

### 2.8 Historical population demography of nuclear DNA

We further estimated rates and directionality of gene flow from the ddRAD‐Seq data with the program *∂a∂i* (Gutenkunst, Hernandez, Williamson, & Bustamante, 2009, 2010). *∂a∂i* implements an efficient diffusion approximation‐based approach to test empirical data against specified evolutionary models (e.g., isolation‐with‐migration). Using *∂a∂i*, a site frequency spectrum was derived from all biallelic RAD‐Seq autosomal SNPs. Loci were concatenated, and SNPs extracted and formatted for *∂a∂i* using the custom python script *nex_mo.py* (Lavretsky et al., 2016). Because we lacked an outgroup, site frequency spectrum data were folded, with only minor alleles considered in the frequency spectrum. Variants observed in zero or in all samples were ignored (masked), as described by Gutenkunst, Hernandez, Williamson, and Bustamante (2010). Finally, for *∂a∂i* to accommodate missing data and differences in sample sizes between Peruvian (*n* = 10 individuals or 20 alleles) and Argentine (*n* = 9 individuals or 18 alleles) torrent duck populations, datasets were projected down to a total of 18 alleles per subspecies. We tested the empirical data against an isolation‐with‐migration evolutionary model as implemented in *∂a∂i* (Gutenkunst, Hernandez, Williamson, & Bustamante, 2009; Gutenkunst et al., 2010). Demographic parameters were estimated, including population sizes (*n_i _*= (*N_i_*/*N*_ref_)*N_Anc_; *N*_ref_ = reference effective population size; *N*_Anc_ = ancestral effective populations size), migration rates (*M_i_*_←_ *_j_* = 2*N*_Anc_ *m_i_*_←_ *_j_*), and divergence times (*t* = *T*/2*N*_ref_ *: T* = time since divergence in generations).

To convert the parameter estimates from *∂a∂i* to biologically informative values, we estimated generation time (*G*) and mutation rates (μ, per locus). First, generation time (*G*) was calculated as *G* = *α* + (*s*/(1 − *s*)), where α is the age of maturity and *s* is the expected adult survival rate (Sæther et al., 2005). Although sexually active by the first generation, most duck species reach sexual maturity in their second year (*α* = 2) with an average adult survival rate of 0.37 estimated for torrent ducks from mark‐recaptured data of the Chillón River (pers. obs.). Together, we estimated a generation time to be 2.5 years. Next, to obtain a mutation rate for nuclear genes, we multiplied a rate of 1.2 × 10^–9^ substitutions/site/year, previously calculated for nuclear genes in other ducks (Peters, Zhuravlev, Fefelov, Humphries, & Omland, 2008) by generation time to attain a rate of 3 × 10^–9^ substitutions site^−1^ generation^−1^ (s s^−1^ g^−1^). A final mutation rate was calculated as the product of the above mutation rate and the total number of base pairs.

## 3 RESULTS

### 3.1 Genetic diversity & population structure—mtDNA

Nucleotide (π/site) and haplotype diversities of the mtDNA CR differed among riverine populations and countries (Table 2). *M. a. leucogenis* in Peru had higher nucleotide diversity that was five times greater than *M. a. armata* in Argentina, although the haplotype diversity was similar between countries (Table 2). These genetic diversity differences suggest a deeper coalescent history in the Peruvian subspecies, while possibly the Argentine subspecies’ shallower coalescence is a result of either demographic or selective processes that determine the lower genetic diversity.

**Table 2:** Nucleotide and haplotype diversity for mtDNA control region (Chillón River, *n* = 57; Pachachaca River, *n* = 49; Arroyo Grande River, *n* = 33 and Malargüe River, *n* = 17), autosomal and Z‐linked markers (Peru‐Chillón, *n* = 10 and Argentina‐Malargüe, *n* = 9) from four populations and two subspecies of torrent duck (*Merganetta armata*) in the Andes of Peru and Argentina

	Mitochondrial haplotypes	Nucleotide diversity (π/site)			Haplotype diversity		
		Mitochondrial	Autosomal	Z Chromosome	Mitochondrial	Autosomal (*SD*)	Z Chromosome (*SD*)
PERU	16	0.015	0.0011	0.0004	0.836	0.139 (0.26)	0.057 (0.15)
Chillón River	">7	0.008			0.543		
Pachachaca River	">10	0.015			0.868		
ARGENTINA	">11	0.003	0.0007	0.0003	0.837	0.093 (0.21)	0.033 (0.13)
Arroyo Grande River	">8	0.004			0.784		
Malargüe River	">4	0.001			0.484		

Abbreviation: SD, standard deviation.

A deep genetic distance corresponding to 4.1% uncorrected mtDNA CR divergence was observed between populations in Peru and Argentina, defining two monophyletic lineages. Specifically, among the four independent rivers we recovered 27 haplotypes characterized by 53 variable sites. Moreover, 16 private haplotypes were found in Peruvian rivers (Chillón and Pachachaca), and 11 private haplotypes were found in Argentine rivers (Arroyo Grande and Malargüe) (Figure 1a; Table S1), resulting in a relative differentiation (Φ_ST_) of 0.82 and consistent with their current subspecies designations (Figure 1c; Table 3). Within Peru or Argentina, we found evidence of strong population structure between the two riverine populations in Peru (Φ_ST PERU_ = 0.43; Figure 1c), as well as between the two rivers in Argentina (Φ_ST ARGENTINA_ = 0.31; Figure 1c). First, we recovered a single mtDNA haplotype that was shared by torrent ducks from the Peruvian Pachachaca and Chillón rivers, whereas those from the Pachachaca River had the highest haplotype diversity and number of private haplotypes of all sampled rivers (Figure 1a; Table 2, Table S1). In Argentina, we once again found a single mtDNA haplotype shared between torrent ducks from the Arroyo Grande and Malargüe rivers, with the Arroyo Grande River having a higher number of private haplotypes as compared to those from the Malargüe River (Figure 1a; Table 2, Table S1). Despite recovered structure among torrent duck populations (Table 3), the relative differentiation within each river population was low (Φ_ST_ < 0.05; Figure 1b) and did not find that mtDNA haplotype frequencies segregated by elevation on the Chillón River (Φ_ST_ = 0.045) and Arroyo Grande River (Φ_ST_ = −0.037), considering a 2,500 m divide along the rivers, or on the Pachachaca River system (Φ_ST_ = 0.029).

**Table 3:** Pairwise Φ_ST_ values (all *p*‐values <.0001) and Tajima's *D* for mtDNA control region (Chillón River, *n* = 57; Pachachaca River, *n* = 49; Arroyo Grande River, *n* = 33 and Malargüe River, *n* = 17), autosomal and Z‐linked markers (Peru‐Chillón, *n* = 10 and Argentina‐Malargüe, *n* = 9) from four populations and two subspecies of torrent duck (*Merganetta armata*) in the Andes of Peru and Argentina

	Φ_ST_ a			Tajima's *D*		
	Mitochondrial	Autosomal	Z chromosome	Mitochondrial (*p*‐value)	Autosomal (*p*‐value)	Z chromosome (*p*‐value)
PERU	0.82	0.3	0.45	1.18 (.91)	−1.25 (.2)	−1.04 (.3)
ARGENTINA				−0.29 (.43)	−1.3 (.19)	0.09 (.93)
Chillón River	0.43			0.13 (.6)		
Pachachaca River				1.00 (.85)		
Arroyo Grande River	0.31			−0.28 (.42)		
Malargüe River				−1.38 (.07)		
Chillón River	0.89					
Any Argentine river						
Pachachaca River	0.84					
Any Argentine river						

a Distance method: Tamura and Nei (1993).

### 3.2 Genetic diversity & population structure—ddRAD‐seq loci

For ddRAD sequencing, a total of 22,662,951 raw reads were recovered from the HiSeq Illumina run of 10 Peruvian and 9 Argentine torrent duck samples. After quality filtering, we recovered 4,216 ddRAD‐seq loci, with 4,027 loci (505,475 base pairs) assigned to autosomes and 189 loci to the Z chromosome (24,242 base pairs); two gametologs were also identified and excluded from analyses. On average, there was a median depth of 283 sequences per individual per locus (range = 18–2,423 sequences/individual/locus).

**Figure 2:**
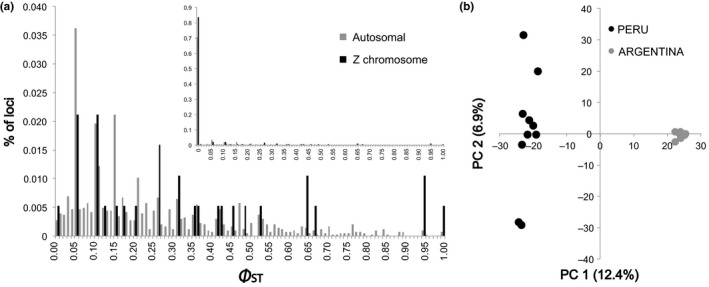
(a) Frequency distribution of Φ_ST_ estimates >0.01 across 4,027 autosomal and 189 Z loci between Peru and Argentina. Inset provides the whole view of the frequency distribution of Φ_ST_. (b) Scatter plot of the first two principal components for 4,027 autosomal ddRAD‐seq markers of torrent duck (*Merganetta armata*) from Chillón River in Peru (*M. a. leucogenis, n* = 10) and Malargüe River in Argentina (*M. a. armata, n* = 9)

First, we recovered a higher autosomal and Z chromosome nucleotide diversity (on average 1.4‐fold higher) and haplotype diversity (on average 1.6‐fold higher) in *M. a. leucogenis* of Peru as compared to *M. a. armata* of Argentina (Table 2). Considering a genomic perspective, the overall Φ_ST_ across all ddRAD‐Seq loci was 0.30 between the two torrent duck subspecies, with higher estimates at Z‐linked (Φ_ST_ = 0.45) than for autosomal (Φ_ST_ = 0.30) markers (Table 3; Figure 2a). A single biallelic SNP was randomly chosen across loci, with a total of 2,356 and 49 biallelic autosomal and Z‐linked SNPs, respectively, used for ADMIXTURE analyses. The optimal *K* was two for both autosomal and Z‐linked markers, and ADMIXTURE results largely distinguished between the two subspecies with 99% of assignment probabilities and were concordant with the PCA results, plotted with the first two principal components, in which the two subspecies were differentiated in two discrete clusters, whereas individuals from Peru are much more dispersed along PC1 than the individuals from Argentina (Figure 2b; Figure S1). No additional structural resolution was attained when analyzing higher values of *K*.

### 3.3 Historical population demography

**Figure 3:**
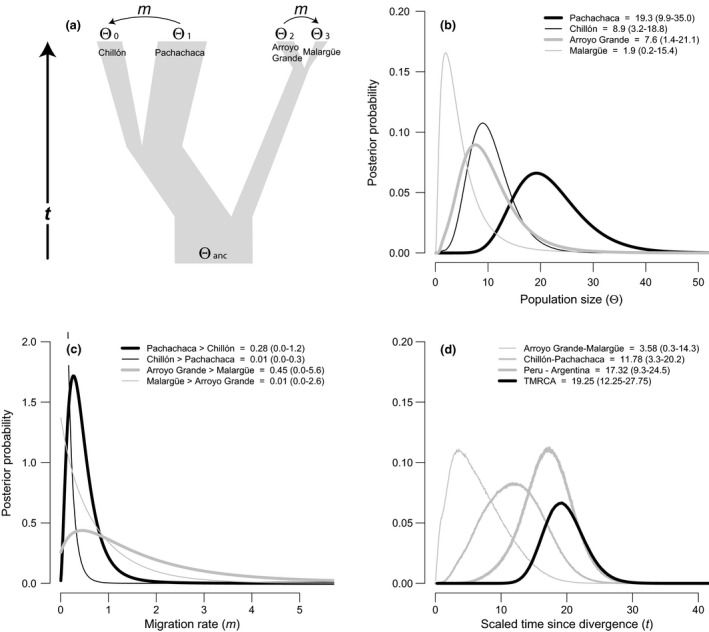
(a) Isolation‐with‐migration model for two sampled populations of Peruvian torrent duck (*M. a. leucogenis*) in Pachachaca and Chillón, and two populations of Argentine torrent duck (*M. a. armata*) in Arroyo Grande and Malargüe, and posterior probability distribution graphs (b, c and d) for historical demographic parameters estimated using IMa2 analysis in a pairwise comparison of localities. Height of the curves corresponds to the estimated probability that a given parameter value is true, given the data (95% confidence intervals are reported in each graph). (b) Effective population sizes (Θ) scaled to the neutral mutation rate estimates for each one of the four localities sampled. (c) Scaled migration rates (*m*) between four pairs of localities. (d) Scaled time since divergence (*t*) estimates between three pairs of localities and the scaled time to most recent common ancestor (TMRCA)

**Figure 4:**
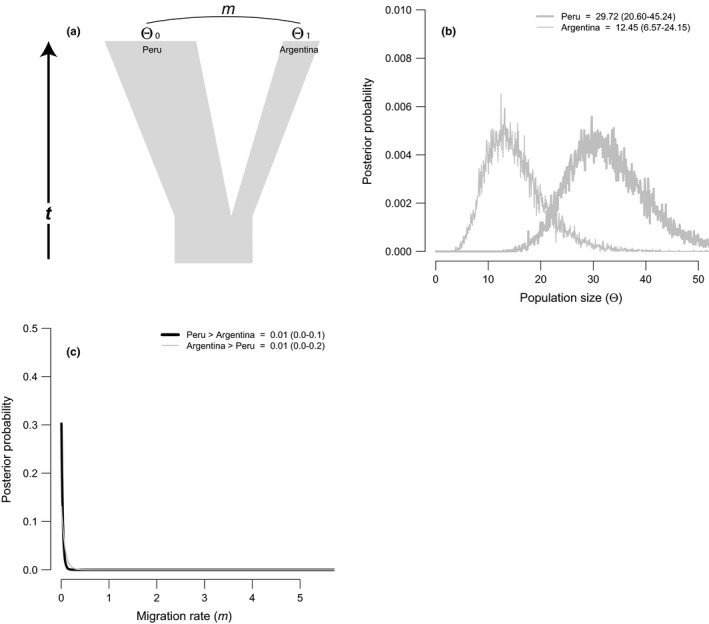
(a) Isolation‐with‐migration model for two torrent duck (*Merganetta armata*) subspecies in the Andes of Peru and Argentina and posterior probability distribution graphs (b and c) for historical demographic model parameters estimated using IM analysis in pairwise comparisons. Height of the curves corresponds to the estimated probability that a given parameter value is true, given the data (95% confidence intervals are reported in each graph). (b) Effective population sizes (Θ) scaled to the neutral mutation rate estimated for each country and ancestral population size. (c) Scaled migration rates (*m*) between two pairs of countries

The effective population size parameters, Θ, scaled to the neutral mutation rate in IM and IMa2 for mtDNA were higher for the rivers in Peru than Argentina (Figures 3b and 4b). The largest Θ was for the Pachachaca River with a peak at 19.3 (95% HPD = 9.9–35.0; Figure 3b) in Peru; the smallest Θ was an order or magnitude lower on the Malargüe River with a peak at 1.9 (95% HPD = 0.2–15.4; Figure 3b). Between subspecies, the Peruvian torrent duck population was estimated to have the largest overall effective population size (29.72, 95% HPD = 20.60–45.24; Figure 4b) compared to the Argentine population (12.45, 95% HPD = 6.57–24.15; Figure 4b). As to be expected for mtDNA, because the two clades were reciprocally monophyletic, the ancestral population sizes estimated with IM and IMa2 were flat and did not show clear peaks (posterior probabilities not shown in the figures).

The *∂a∂i* analyses utilizing the genomic data in the isolation‐with‐migration model provided results that agree the mtDNA‐estimated results. Using autosomal markers, we estimated an averaged mutation rate of 1.52 × 10^–3^ s s^−1^ g^−1^ (3 × 10^–9^ s s^−1^ g^−1^ × 505,475 base pairs) that was used to convert *∂a∂i* results. Given the isolation‐with‐migration model, parameter estimates suggested different effective population sizes (*Ne*) for the two subspecies of torrent ducks (*N*_e PERU_ = 49,896 and *N*_e ARGENTINA_ = 19,008).

Tajima's *D* values for mtDNA for the populations in Argentina and Peru (Table 3) were not significantly different from zero (higher *p‐values*). Therefore, we did not observe a significant excess of rare nucleotide site variants compared to what would be expected under a neutral model of evolution, and each riverine population may be evolving as per mutation‐drift equilibrium. In contrast, Tajima's *D* values were negative for autosomal and Z‐linked markers for both subspecies populations in Peru and Argentina, although both markers had not statistically significant *p‐values* (Table 3).

Estimating gene flow, the four‐population IMa2 analysis suggests that the number of migrants (*m*) between riverine populations within each country is effectively low and unidirectional (Figure 3c). Importantly, we were unable to reject the hypothesis of no gene flow. The posterior distribution of *m* (scaled effective migration rate relative to the mutation rate, *m*/μ) within Peru peaked at 0.28 from the larger Pachachaca population to the smaller Chillón population (95% HPD = 0.0–1.2; Figure 3c), and in the case of Argentina peaked at 0.45 from the larger Arroyo Grande population to the smaller Malargüe population (95% HPD = 0–5.6; Figure 3c). By contrast, we failed to reject the hypothesis of no gene flow in the opposite directions between rivers within each country. Moreover, the IM analysis between the subspecies suggests that the number of migrants between Peru and Argentina is not different from zero (0.01, 95% HPD = 0.0–0.1; Figure 4c). However, the *∂a∂i* results of the nuclear data supported slightly greater, yet significant gene flow into *M. a. armata* in Argentina from *M. a. leucogenis* in Peru (2*Nm*_21_ = 0.30 migrants/generations) as compared to gene flow from *M. a. armata* into *M. a. leucogenis* (2*Nm*_21_ = 0.21 migrants/generations).

For time since divergence, the posterior probability distribution of *t* (scaled time since divergence) with the IMa2 analysis between the two torrent duck subspecies peaked at 17.32 (95% HPD = 9.3–24.5; Figure 2d). This value converted to time in years (Peters et al., 2005) suggests that Peruvian and Argentine torrent ducks subspecies began diverging about 600,000 years before present (ybp) (95% HPD range = 1.2 Mya to 200,000 ybp). TMRCA peaked at 19.25 (95% HPD = 12.24–27.75; Figure 2d), indicating that all sampled haplotypes coalesce at approximately 650,000 ybp. (95% HPD range = 1.4 Mya to 280,000 ybp). Converting the *∂a∂i* results of the nuclear data also indicates divergence within the same approximate timeframe (782,490 ybp).

## 4 DISCUSSION

We provide the most comprehensive molecular analysis of torrent ducks to date and clearly identify limited or no gene flow and strong differentiation across the mitochondrial and nuclear genomes of torrent ducks found in the rivers of Peru versus Argentina. These results support the current taxonomic subspecies designation of Peruvian and Argentine torrent ducks. Moreover, we are able to conclude that these two subspecies probably originated during the early‐ to mid‐Pleistocene as a result of an old divergent nonvicariant event subsequent to the origin of the South American Arid Diagonal phylogeographic barrier (Burniard, 1982). Finally, we also recovered strong population structure within each country that is probably explained by both a strong territoriality and breeding site fidelity in adults, and the geographic isolation by distance between riverine subpopulations.

### 4.1 Genetic diversity and small effective population size

In general, diversity estimates across mtDNA haplotypes for the two torrent ducks subspecies were higher or similar to those found in other Andean duck species (Table 2; Bulgarella et al., 2012; Wilson, Peters, & McCracken, 2012). Moreover, despite the constraint on *Ne* due to their exclusive preference for torrential riverine (linear) habitats (Ellegren & Galtier, 2016; Moffet, 1970), torrent ducks have higher mtDNA diversity estimates as compared to other torrential riverine specialist birds (e.g., New Zealand endemic blue ducks (*Hymenolaimus malacorhynchos*; Grosser, Abdelkrim, Wing, Robertson, & Gemmell, 2016); white‐throated dipper (*Cinclus cinclus*; Hourlay et al., 2008)). In fact, torrent ducks from Peru had values of mtDNA diversity more similar to those found in other ducks with *Ne* several orders of magnitude larger *Ne* (e.g., mallards; Lavretsky et al., 2015). Therefore, because mutation rates are likely to be similar across duck species, we can hypothesize that torrent duck mtDNA genetic diversity probably is influenced by the structure of their habitat leading to extensive branched and subdivided, structured riverine habitat, such that each river may be isolated by distance and genetically distinct from other watersheds. In contrast to mtDNA, diversity estimates from nuclear DNA for the two torrent duck subspecies were lower compared to other waterfowl species (Lavretsky et al., 2015; Lavretsky et al., 2016; Peters et al., 2016; Wilson et al., 2012). A probable explanation for the disparity in diversity estimates of mitochondrial and nuclear genes within torrent ducks could be a combined effect resulting from high mtDNA mutation rate (four orders of magnitude), population subdivision due to the branched structure riverine habitat, the adult territorial behavior, and natural selection associated to high elevation (e.g., low temperatures and hypoxia) affecting nuclear DNA.

Finally, between countries (Argentina vs. Peru) and within countries (e.g., Chillón River vs. Pachachaca River), we found that populations inhabiting shorter rivers compared to populations inhabiting longer or branched rivers systems had the lowest levels of molecular diversity (Figure 1; Tables 1 and 2). This low genetic diversity associated with river length is more evident in the southern latitudinal rivers sampled in Argentina, which may have experienced founder events following glaciation periods. In general, these rivers are shorter than the Peruvian rivers and more likely maintaining small *Ne* that might be more susceptible to loss of diversity due to genetic drift (Table 1; Figure 3b). Moreover, current and historical changes in the river water regimes due to extreme drought and dramatic climate changes (Rivera, Penalba, Villalba, & Araneo, 2017) can produce bottlenecks, or riverine population extinctions. These events can establish recurrent population extinction–colonization or metapopulation dynamics that would reduce *Ne* and extend the effects of genetic drift. Variable and small estimates of *Ne* across riverine populations, as well as mostly negative Tajima's *D* (i.e., population growth), although not significant, possibly suggest a metapopulation dynamic. Specifically, our findings for Argentine populations prompt that these likely have been smaller and possibly have gone through multiple extinction–colonization events, whereas Peruvian populations have likely been large and resident to rivers that have had a prolonged period of water regime stability as compared to those in Argentina (Figures 3b and 4b; Table 3).

### 4.2 Deep genetic divergence between subspecies

We found mtDNA monophyly and nonsignificant estimates of gene flow between the two torrent duck subspecies, *M. a. leucogenis* and *M. a. armata* (Figures 1a and 4a,c; Table 3; also for nuclear DNA Figure 2b, Figure S1). The reduced gene flow between the two torrent ducks subspecies is most likely due to their habitat specialization and lack of suitable intermediate habitat, particularly where the Andes show the highest mountain ranges and widest extension that harbors a complex riverine spatial structure (Capitonio et al., 2011; Gonzalez & Pfiffner, 2011). In contrast, two subspecies of speckled teal (*Anas flavisrostris*) with similar distribution range and divergence time show relatively less genomic divergence and asymmetric gene flow (Graham et al., 2017). Long‐standing and recurrent gene flow in speckled teal might be facilitated by the lack of habitat specialization (i.e., lakes, pond, and also rivers), and a larger number of habitats present in the lowlands due to a broader flat topography than in the highlands in the Southern Andes. Therefore, when and where geographic barriers isolate these torrent duck subspecies is likely tightly connected with the orogeny of the Andes, and the formation and persistence of torrential riverine systems (Fjeldså, 1985).

Finally, we estimate that the two torrent ducks likely diverged in the early‐ to mid‐Pleistocene (mtDNA divergence time = 600,000 ybp, 95% HPD range = 1.2 Mya to 200,000 ybp, Figure 3d; and nuclear divergence time = 782,490 ybp). Compared to other species found in the Andes of Argentina and Peru, divergence estimates for torrent ducks are some of the oldest, although comparable to those estimated between speckled teal (Bulgarella et al., 2012; Graham et al., 2017; McCracken et al., 2009; Wilson et al., 2012). Therefore, torrent ducks can be considered an older Andean resident probably tightly associated to the formation of river systems. However, divergence of these two subspecies likely followed the establishment of all major river systems as the Andes started to rise during the Late Cretaceous (~60–80 Mya) and were at their final heights by the Early Pliocene (~3–4 Mya) (Capitonio et al., 2011; Clapperton, 1983; Garzione et al., 2008; Hartley, 2003), which are well before estimated divergence times.

### 4.3 South American Arid or Dry Diagonal

Historical and current field records show that two dry Andean regions serve as a disjunction between the distribution ranges of *M. a. leucogenis* and *M. a. armata*, probably due to the lack or extreme seasonality of torrential rivers in these regions (Conover, 1943; eBird, 2016; Fjeldså & Krabbe, 1990; Johnsgard, 2010; Johnson, 1963). One of these regions is located in the junction (above 28°S) of both the Atacama (Chile) and Monte Deserts (Argentina), hyper arid and arid systems, respectively. A second region (below 18°S) is also located in the dry Puna in the Eastern Cordillera in Southern Bolivia. Both regions are part of the South American Arid or Dry Diagonal that traverses the continent from the North Coast of Peru to the Argentine Patagonia. Believed to have originated with the orogeny and establishment of the Andes Mountains and reinforced by old and recent glaciations (Blisniuk, Stern, Chamberlain, Idleman, & Zeitler, 2005; Hartley, 2003; Rabassa, Coronato, & Martínez, 2011; Rabassa, Coronato, & Salemme, 2005; Zachos, Pagani, Sloan, Thomas, & Billups, 2001), the Arid Diagonal is defined by its dry climate and absence of glaciers, a defining feature dividing the Central Andes from Southern Andes and Patagonia (Figure 1c; and Burniard, 1982; Seltzer, 1990). Thus, the Arid Diagonal is likely a barrier to movement for torrent ducks as it has been for a variety of other birds (aquatic and terrestrial; Fjeldså, 1985; Ridgely & Tudor, 2009; Valqui, 2008; Voelker, 2002; Vuilleumier, 1998), mammals (González, Samaniego, Marín, & Estades, 2013; Marin et al., 2007), insects (habitat of endemisms; Roig‐Juñent, Flores, Claver, Debandi, & Marvaldi, 2001), and plants (Chacón, Camargo de Asis, Meerow, & Renner, 2012; Martins, Scherz, & Renner, 2014; Murillo‐A, Stuessy, & Ruiz, 2016; Quiroga, 2010) found in South America.

Moreover, given that these historical climate conditions of the Arid Diagonal likely reduced the river habitat suitability long before the estimated time since divergence between these two torrent duck subspecies, we hypothesize that colonization through dispersal and subsequent isolation likely best explains their divergence. Although rare, torrent ducks are known to make long distance movements (Cerón & Capllonch, 2016) that would permit them to traverse across large landscapes like the Dry Diagonal. In general, we hypothesize that it is very likely that these two subspecies were probably originated by an old divergence through multiple founder events, with individuals dispersing from the Central Andes to the Southern Andes, because the large *Ne* and genetic diversity recovered in Peru compared to Argentina, during the early‐ to mid‐Pleistocene.

### 4.4 Population structure among rivers, “extended family” within rivers and metapopulation

Based on mitochondrial DNA, we estimated low or zero gene flow between rivers within each country, suggesting well‐differentiated and moderately structured river populations in Peru and Argentina, respectively. These results indicate that each river system, inside of a watershed, contains a particular group of genetically more similar individuals. We hypothesize that these results are likely due to three nonmutually exclusive hypotheses: (1) Strong territoriality and breeding site fidelity in adults increase the similarity of the individuals within a river, (2) geographic (e.g., watershed boundary, high mountain ranges) or climatic barriers (e.g., large deserts) isolate rivers preventing the dispersal of the individuals, and driving an independent evolution of each riverine population by genetic drift, and (3) individual dispersal capacity (i.e., frequency and distance) describes an isolation‐by‐distance pattern that in short distance dispersal resembles a metapopulation model. For example, the year‐round territorial behavior, the long‐term pair bonds, and the strong site fidelity in adult torrent ducks, as mentioned in the first hypothesis, can prevent gene flow among rivers, but also contribute to the similarity of the individuals of a growing population in vacant or improved habitat, and thus increase the relatedness within each riverine population and develop an “extended family” (Alza et al., 2017; Cardona & Kattan, 2010; Eldridge, 1986; Hartl & Clark, 2007; Pernollet, Estades, & Pavez, 2012). Additionally, as referred in the second hypothesis, torrential river systems inside watersheds are analogous to islands (islands on mountain slopes) that isolate and sustain populations and communities (Black, 1997; Naiman, Magnuson, McKnight, Stanford, & Karr, 1995; Omernik & Bailey, 1997; Sullivan, Watzin, & Keeton, 2007), similarly observed in lakes (Barbour & Brown, 1974), marshes (Brown & Dinsmore, 1988), caves (Culver, Holsinger, & Baroody, 1973), mountaintops (Nores, 1995), or woodlots (Holland, 1978). Thus, as new rivers are colonized (e.g., by a small group of individuals or for a long term), these isolated populations may behave independently of the other or larger population, growing as distinct populations and leading to increased population structure among rivers (Mayr, 1963; Phillimore & Owens, 2006). Finally, it is possible that different watershed (sub)‐populations may be genetically more connected or completely differentiated depending on the distance among them and the dispersal capacity of the ducks. Among torrent duck riverine (sub)‐populations it appears that dispersal is most likely to occur at close range (Cerón & Capllonch, 2016; Paradis, Baillie, Sutherland, & Gregory, 1998), which could also reduce their differentiation and resemble a metapopulation model (Hanski & Gaggiotti, 2004; Levins, 1968). Similar population structure patterns have been described in the blue duck (*Hymenolaimus malacorhynchos*) in New Zealand with a regional genetic differentiation (region around 100 km) associated with isolation‐by‐distance pattern (Grosser et al., 2016). To test these hypotheses, future work would benefit from measures of relatedness on increased sampling of individuals across rivers, including multiple adjacent river systems stratified by elevation. We note that complete haplotype admixture (mtDNA) along each study river system suggests that torrent ducks are not segregated by elevation (considering a 2,500 m divide, Figure 1b). Previous studies have reported seasonal altitudinal movements of torrent ducks, and these movements can be influenced by reproductive season, food availability, variation in the streamflow, and winter conditions, especially in the Southern Andes (Johnsgard, 1966; Johnson, 1963; Pernollet et al., 2012; Ramírez, Botero, & Kattan, 2014). Also during our banding activities in the Chillón River, we recovered two individuals that had moved at least 4 km along the river. These direct observations of altitudinal movements corroborate the absence of population structure along elevation gradient within each river. Thus, the physiological restriction imposed by hypoxia at elevations >2,500 m appears to not constrain mitochondrial gene flow between low‐ and high‐altitude localities on any of the rivers we sampled, despite recently reported elevational variation in body size (Gutiérrez‐Pinto et al., 2014), function of key enzymes (Dawson et al., 2016), changes in insulative properties of feathers (Cheek et al., 2018), and changes in the hypoxic ventilatory response (Ivy et al., 2019).

### 4.5 Conservation relevance

Torrent ducks are vulnerable to systematic pressure and stochastic perturbations because of their specialization to whitewater streams, low population densities, patchy distribution, low reproductive potential, and reliance on water quality and aquatic insect populations (Alvarez, Astie, Debandi, & Scheibler, 2014; Carboneras, 1992; Shaffer, 1981; Zwick, 1992). Indeed, populations living in rivers and streams habitats are susceptible to extinction as a result of the high vulnerability that freshwater systems have pollution, siltation, and anthropogenic disturbance. Thus, torrent ducks had suffered local extinction and decreasing population trends of many river populations (Callaghan, 1997; Cerón & Trejo, 2012; Pernollet, Pavez, & Estades, 2013; Weller, 1975). In particular, the recognized causes of their riverine extinction are as follows: predation on offspring by exotic invasive species (mink; Cerón & Trejo, 2012), competition for aquatic insect food sources (trout; Eldridge, 1986), loss of territories and habitats due to urbanization and contamination of rivers in Peru and Colombia (J. M. Barbarán, personal communication; Cardona & Kattan, 2010), management of the watershed to provide drinking water, irrigation and hydropower production for mining activities and human communities (Pernollet et al., 2013), and hunting without proper regulation (pers. obs.). Whereby, the three subspecies regionally had been classified under threatened categories (Vulnerable and Endangered) (Cerón & Trejo, 2012; Green, 1996), even though the overall species is still classified as a “Least Concern” (BirdLife International, 2016). Our study also emphasizes the necessity to protect and manage the subspecies and riverine populations as separate conservation units due to the strong genetic structure and very low gene flow. Therefore, the establishment of monitoring programs of the subspecies and multiple populations is a priority to evaluate their metapopulation structure and understand the actual status of the species (Phillimore & Owens, 2006), even more, under the threat of deglaciation and regional drought conditions associated to climate change in the Andes. In general, the torrent duck provides an excellent opportunity to examine the patterns and the mechanisms related to strong population structure among small and isolated populations, and these results can be contrasted with other riverine ducks, such as the African black duck (*Anas sparsa*), and Salvadori's duck (*Salvadorina [Anas] waigiuensis*) in New Guinea, or habitat specialist species.

## CONFLICT OF INTEREST

None declared.

## AUTHOR CONTRIBUTIONS

L.A. analyzed the data and drafted the manuscript; L.A., M.S., C.K., and K.G.M. collected and acquired the data; P.L. acquired and analyzed the genomic data and helped to revise the manuscript; L.A. and G.C. conceived the ideas; J.P. interpreted the results and revised the manuscript; K.G.M. designed the study, interpreted the results, and helped to write the manuscript; and all the authors have read and commented on the final manuscript.

## Data Availability

DNA sequences: GenBank (Accession Numbers MN196318:MN196473). Raw Illumina reads: NCBI's Sequence Read Archive (SRA data: SRP215991).
