# Supplementary material for: Old divergence and restricted gene flow between torrent duck (Merganetta armata) subspecies in the Central and Southern Andes
Source: Ecol Evol. 2019 Aug 15;9(17):9961–76. doi: 10.1002/ece3.5538 (PMC6745679; doi:10.1002/ece3.5538)
Supplement: Supplementary file 1 [file ECE3-9-9961-s001.docx]

**Old Divergence and Restricted Gene Flow Between Torrent Duck (*Merganetta armata*) Subspecies in the Central and Southern Andes**

**Supplementary information**

Luis Alza, Philip Lavretsky, Jeffrey L. Peters, Gerardo Cerón, Matthew Smith, Cecilia Kopuchian, Andrea Astie, and Kevin G. McCracken

**TABLE S1** Distribution of the frequencies of the 27 haplotypes by populations (rivers) and country found for the two torrent duck (*Merganetta armata*) subspecies in the Andes of Peru and Argentina. No haplotypes were shared between countries.

| Watershed population / Haplotype | PERU (*n* = 106) | | ARGENTINA (*n* = 50) | |
| --- | --- | --- | --- | --- |
|  | Chillón River | Pachachaca River | Arroyo Grande River | Malargüe River |
|  | (*n* = 57) | (*n* = 49) | (*n* = 33) | (*n* = 17) |
| 1 | 36 | 0 | 0 | 0 |
| 2 | 14 | 0 | 0 | 0 |
| 3 | 2 | 0 | 0 | 0 |
| 4 | 1 | 0 | 0 | 0 |
| 5 | 2 | 0 | 0 | 0 |
| 6 | 1 | 0 | 0 | 0 |
| 7 | 1 | 6 | 0 | 0 |
| 8 | 0 | 4 | 0 | 0 |
| 9 | 0 | 1 | 0 | 0 |
| 10 | 0 | 1 | 0 | 0 |
| 11 | 0 | 11 | 0 | 0 |
| 12 | 0 | 11 | 0 | 0 |
| 13 | 0 | 5 | 0 | 0 |
| 14 | 0 | 5 | 0 | 0 |
| 15 | 0 | 4 | 0 | 0 |
| 16 | 0 | 1 | 0 | 0 |
| 17 | 0 | 0 | 1 | 0 |
| 18 | 0 | 0 | 4 | 0 |
| 19 | 0 | 0 | 2 | 0 |
| 20 | 0 | 0 | 1 | 0 |
| 21 | 0 | 0 | 8 | 0 |
| 22 | 0 | 0 | 2 | 0 |
| 23 | 0 | 0 | 3 | 0 |
| 24 | 0 | 0 | 12 | 1 |
| 25 | 0 | 0 | 0 | 13 |
| 26 | 0 | 0 | 0 | 2 |
| 27 | 0 | 0 | 0 | 1 |
| Total by river | 7 | 10 | 8 | 4 |
| Total by country | 16 | | 11 | |

**FIGURE S1** Maximum-likelihood estimation (ADMIXTURE) of individual assignment probabilities for *K* = 2 populations based on autosomal loci (*n* = 10 *Merganetta armata leucogenis* and 9 *M. a. armata*). The results show population structure between the two torrent duck (*M. armata*) subspecies in the Andes of Peru and Argentina.
